# Correlation between white blood cell count and intestinal resection in patients with acute mesenteric vein thrombosis

**DOI:** 10.1186/s12876-024-03172-4

**Published:** 2024-02-23

**Authors:** Yu Xu, Shang-Tai Dai, Hong-Qiao Lu, Wei Chen, Zhi-Wei Xiong, Jiang Liu, Yong-Jiang Tang, Shi-Kui Guo, Kun-Mei Gong

**Affiliations:** 1 Panzhihua Central Hospital, 34 Yikang St, 617000 Panzhihua, Sichuan Province China; 2 The Affiliated Hospital of Kunming University of Science and Technology, No. 157 Jinbi Road, 650500 Kunming City, Yunnan Province P.R. China

**Keywords:** White blood cell count, Acute mesenteric vein thrombosis, Enterectomy

## Abstract

**Objective:**

Acute mesenteric vein thrombosis (AMVT) is an acute abdominal disease with onset, rapid progression, and extensive intestinal necrosis that requires immediate surgical resection. The purpose of this study was to determine the risk factors for nosocomial intestinal resection in patients with AMVT.

**Methods:**

We retrospectively analysed 64 patients with AMVT diagnosed by CTA at the Affiliated Hospital of Kunming University of Science and Technology from January 2013 to December 2021. We compared patients who underwent intestinal resection (42 patients) with those who did not undergo intestinal resection (22 patients). The area under the ROC curve was evaluated, and a forest map was drawn.

**Results:**

Among the 64 patients, 6 (9.38%) had a fever, 60 (93.75%) had abdominal pain, 9 (14.06%) had a history of diabetes, 8 (12.5%) had a history of deep vein thrombosis (DVT), and 25 (39.06%) had ascites suggested by B ultrasound or CT after admission. The mean age of all patients was 49.86 ± 16.25 years. The mean age of the patients in the enterectomy group was 47.71 ± 16.20 years. The mean age of the patients in the conservative treatment group (without enterectomy) was 53.95 ± 15.90 years. In the univariate analysis, there were statistically significant differences in leukocyte count (*P* = 0.003), neutrophil count (*P* = 0.001), AST (*P* = 0.048), total bilirubin (*P* = 0.047), fibrinogen (*P* = 0.022) and DD2 (*P* = 0.024) between the two groups. The multivariate logistic regression analysis showed that admission white blood cell count (OR = 1.153, 95% CI: 1.039–1.280, *P* = 0.007) was an independent risk factor for intestinal resection in patients with AMVT. The ROC curve showed that the white blood cell count (AUC = 0.759 95% CI: 0.620–0.897; *P* = 0.001; optimal threshold: 7.815; sensitivity: 0.881; specificity: 0.636) had good predictive value for emergency enterectomy for AMVT.

**Conclusions:**

Among patients with AMVT, patients with a higher white blood cell count at admission were more likely to have intestinal necrosis and require emergency enterectomy. This study is helpful for clinicians to accurately determine whether emergency intestinal resection is needed in patients with AMVT after admission, prevent further intestinal necrosis, and improve the prognosis of patients.

## Introduction

Although the mesenteric vein accounts for less than 10% of the intestinal blood supply [1], acute intestinal necrosis is relatively common in the clinic. Septic shock caused by acute mesenteric vein necrosis is still one of the most serious complications, regardless of whether the portal vein is involved in thrombosis [2]. In addition, postoperative intestinal stenosis, short bowel syndrome and other complications also seriously affect the prognosis of patients [3]. According to reports, early active anticoagulation after admission can effectively reduce the incidence of intestinal necrosis [4], and anticoagulation has become a first-line treatment to significantly improve the prognosis of patients with mesenteric vein thrombosis, becoming a necessary condition for successful nonsurgical treatment [5]. The prognosis of patients with acute mesenteric thrombosis is different, and the most important factors leading to death of patients are intestinal necrosis, toxin absorption into the blood, septic shock, and finally death. It is generally believed that intestinal necrosis should be considered when there is an obvious peritoneal stimulation sign, bloody ascites found on abdominal puncture, and general deterioration of the body. Laparoscopic exploration and intestinal resection should be performed immediately to stop the development of the disease. The diagnosis and timely surgical treatment of intestinal necrosis are closely related to the prognosis of patients [6]. The aim of this study was to assess risk factors for patients undergoing emergency enterectomy and to improve survival and outcomes in these patients.

## Data and research population

**Fig. 1:**
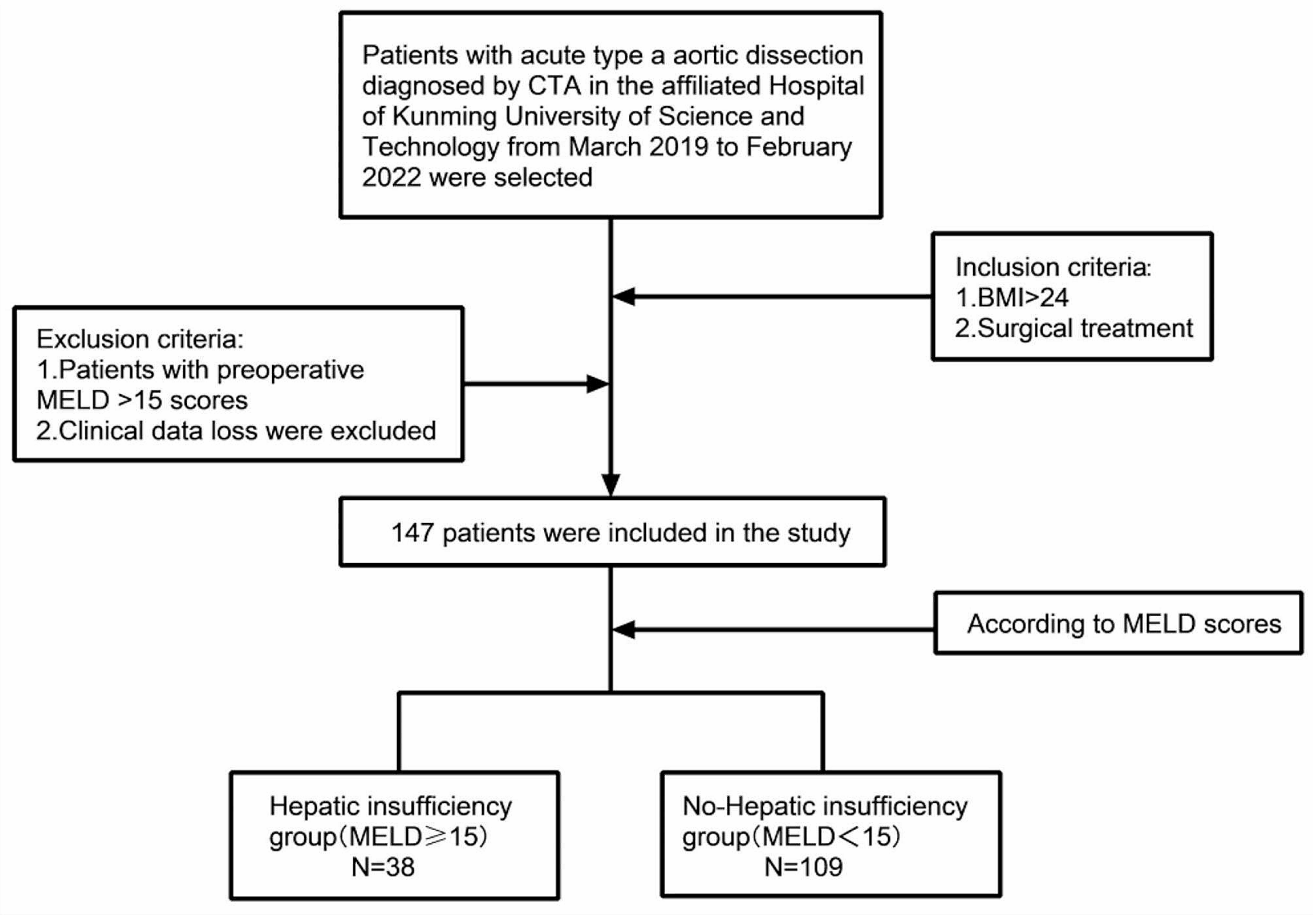
Flow chart

There was a total of 64 patients confirmed by CTA in the Affiliated Hospital of Kunming University of Science and Technology from January 2013 to December 2021, and those who underwent intestinal resection after admission were enrolled as the experimental group (42 patients). A total of 22 patients without intestinal resection (conservative treatment) were used as the control group, the flow chart of the study design is Fig. 1. All patients gave informed consent to the study. The inclusion criteria were as follows: patients confirmed by CTA, and the exclusion criteria was patients with any clinical data loss. This study was approved by the Ethics Committee of the Faculty of Medicine, Kunming University of Science and Technology.

### Data collection

All included patients were divided into the enterectomy group and the conservative treatment group (no enterectomy) according to whether they underwent surgery after admission. The included data included age, fever, abdominal pain after admission, history of hypertension, diabetes, deep vein thrombosis, smoking history, routine blood tests, biochemical tests, and ascites after admission.

## Statistical method

SPSS 22.0 was used for data analysis in this study. A t test was used for variables conforming to the normal distribution, a rank-sum test was used for variables not normally distributed, and the chi-square test and Fisher’s probability method were used for categorical variables. The data with *P* < 0.05 were included in binary logistic regression analysis to obtain the OR value and 95% confidence interval, and a forest map was drawn. An ROC curve was drawn for variables included in the binary logistic regression model to obtain the area under the curve to predict whether patients with mesenteric vein thrombosis need active surgical exploration and enterectomy after admission.

## Result

### Clinical characteristics

There were a total of 64 patients; 42 patients who underwent enterectomy were included in the experimental group, and 22 patients who received conservative treatment (without enterectomy) were included in the control group. The average age of all patients was 49.85 ± 16.25 years old, 47.71 ± 16.20 years old in the surgery group, and 53.95 ± 15.90 years old in the conservative treatment group. Male patients accounted for 73.44%, 31 patients (48.44%) had smoked before admission, 6 (9.38%) had fever after admission, 60 (93.75%) had abdominal pain, 9 (14.06%) had a history of diabetes, and 8 (12.5%) had a history of deep vein thrombosis (DVT). Twenty-five patients (39.06%) were diagnosed with ascites by B-ultrasound or CT after admission. After admission, a total of 38 patients (59.38%) had white blood cell counts higher than the upper limit of normal value, 6 patients (9.38%) had AST levels higher than the upper limit of the normal value, and 10 patients (15.63%) had abnormal creatinine values. The level of fibrinogen in 42 patients (65.66%) exceeded the upper limit of the normal value, and the DD2 level in 58 patients (90.66%) exceeded the upper limit of the normal value (Table 1).

**Table 1:** Basic characteristics of all petients included in the study

	The overall (*n* = 64)	Surgery(*n* = 42)	No surgery (*n* = 22)	*P*
Gender (Male/female)	47/17	29/13	18/4	0.272
Age	49.86 ± 16.25	47.71 ± 16.20	53.95 ± 15.90	0.146
Fever (yes/No)	6/58	5/37	1/22	0.665
Abdominal pain (yes/no)	60/4	39/3	21/1	0.574
Hypertension (yes/No)	8/56	4/38	4/18	0.430
Diabetes (yes/no)	9/55	6/36	3/19	0.630
DVT(yes/no)	8/56	4/38	4/18	0.430
Smoking history (yes/No)	31/33	21/21	10/12	0.730
WBC (×10^9/L)	12.87 ± 7.13	16.73 ± 6.67	9.33 ± 6.76	0.003
Neutrophils (×10^9/L)	9.64(4.46, 14.59)	11.67(8.19, 14.87)	4.43(3.54, 9.62)	0.001
HGB	142(120, 156)	143(120, 156)	142(120, 155)	0.761
PLT	189(130, 253)	189(128, 228)	211(137, 270)	0.458
AST	22(16, 29)	18(14, 29)	24(20, 34)	0.048
ALT	19(14, 32)	19(14, 33)	18(15, 35)	0.635
Total bilirubin	15.4(11.7, 22.9)	16.4(12.6, 27.8)	13.4(10.6, 20.3)	0.047
Direct bilirubin	6.8(4.7, 10.6)	7.7(5.7, 12.8)	6.3(4.3, 7.7)	0.064
Indirect bilirubin	8.8(6.3, 13.0)	9.4(7.0, 13.9)	7.4(5.4, 11.1)	0.066
Cr	70(61, 89)	69(58, 85)	72(64, 104)	0.164
Blood type(A/B/AB/O)	21/8/20/15	12/4/16/10	9/4/4/5	0.337
K+	4.1(3.8, 4.4)	4.1(3.8, 4.4)	4.2(3.7, 4.3)	0.164
Ascites (yes/no)	25/39	12/25	8/14	0.793
FIB	4.4(3.2, 6.2)	5.0(3.9, 6.3)	3.9(3.0, 5.2)	0.022
APTT	38.42 ± 4.66	38.41 ± 4.73	38.43 ± 4.63	0.984
PT	14.5(13.6, 153)	14.7(13.9, 15.4)	13.7(13.5, 15.0)	0.090
DD2	12.3(5.1, 19.3)	14.5(6.5, 21.8)	9.3(2.9, 15.7)	0.024

### Univariate analysis

The univariate analysis showed that leukocyte count (*P* = 0.003), neutrophil count (*P* = 0.001), AST (*P* = 0.048), total bilirubin (*P* = 0.047), fibrinogen (*P* = 0.022), and DD2 (*P* = 0.024) were significantly different between the enterectomy group and the group without enterectomy.

### Multivariate analysis of enterectomy after admission in patients with acute mesenteric vein thrombosis

**Fig. 2:**
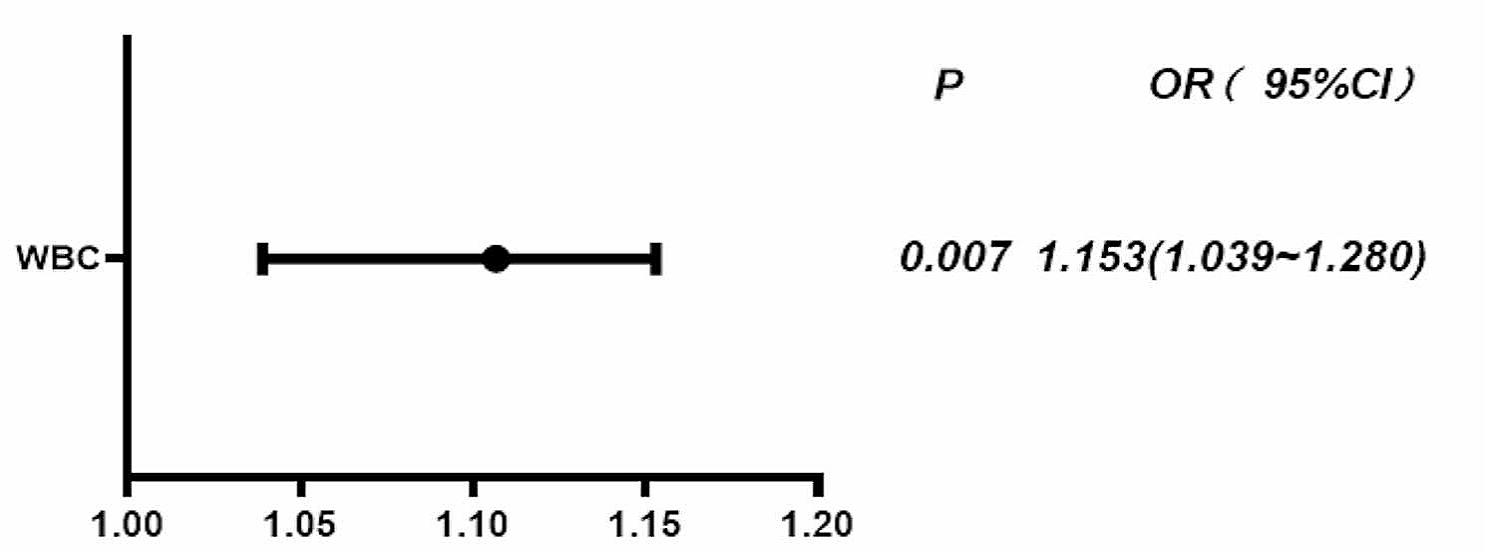
Multivariate logistics analysis -- Forest map

The multivariate logistic regression analysis showed that admission white blood cell count (OR = 1.153, 95% CI: 1.039–1.280, *P* = 0.007) was an independent risk factor for enterectomy in patients after admission (Table 2; Fig. 2).

**Table 2:** Multivariate logistics analysis of emergency enterectomy in AMVT patients

	OR	95%CI	*P*
WBC	1.153	1.039 ∼ 1.280	0.007

### Predictive value of ROC curves and forest map

**Fig. 3:**
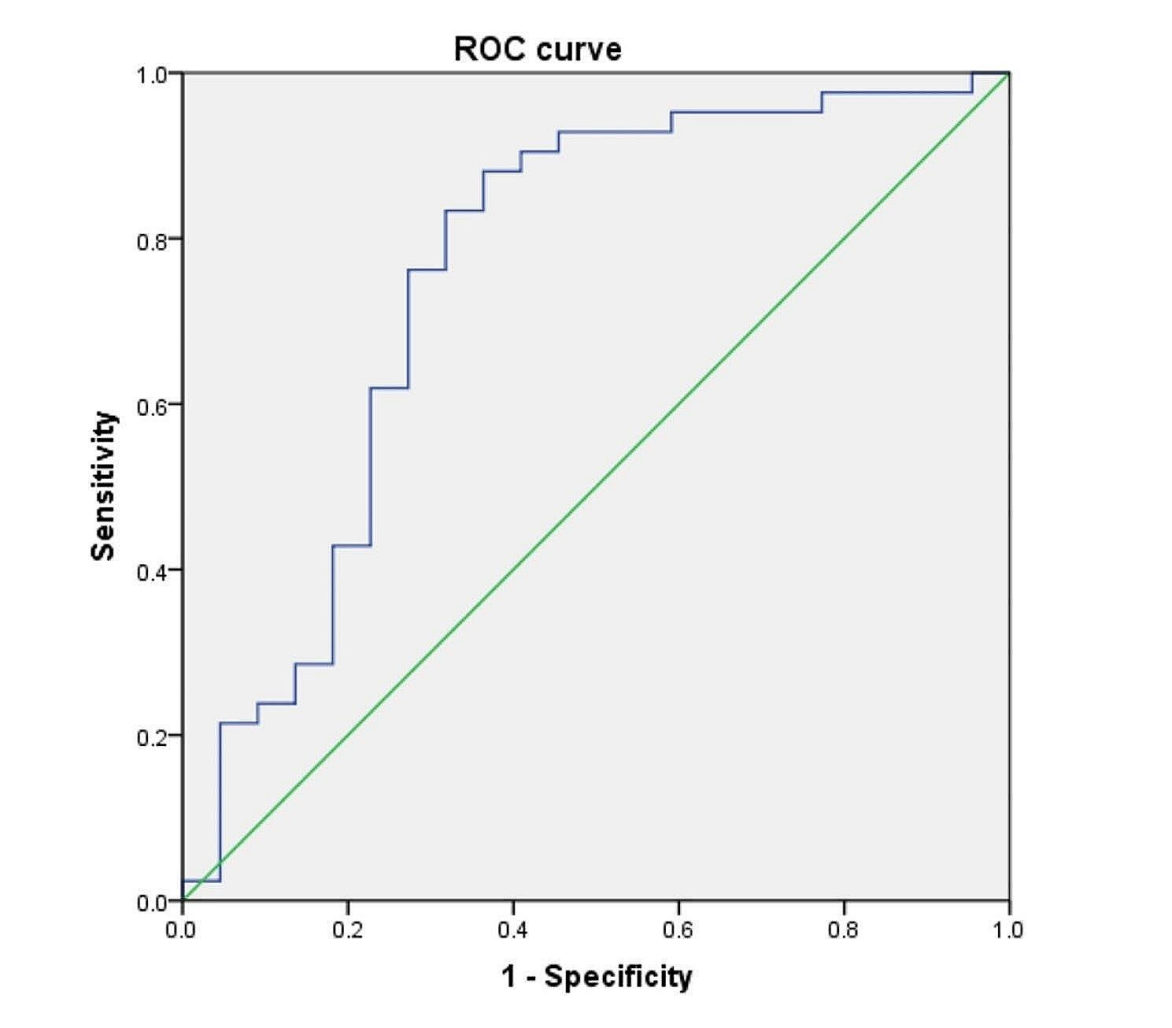
ROC curve of white blood cell count predicts enterectomy at admission in AMVT patients

The ROC curve showed that the AUC of white blood cell count predicting enterectomy for patients after admission was 0.759, the 95% CI was 0.620–0.897, the optimal critical value was 7.815, the sensitivity was 0.881, the specificity was 0.636, and the *P* value was 0.001. The forest plot showed that the OR value of this study was 1.153, 95% CI: 1.039–1.280, and the *P* value was 0.007, indicating that white blood cell count was an independent risk factor for enterectomy in patients after admission (Table 3; Fig. 3).

**Table 3:** ROC curve of white blood cell count predicts enterectomy at admission in AMVT patients

	AUC	95%CI	*P*	Optimum critical value	Sensitivity	Specificity
WBC	0.759	0.620 ∼ 0.897	0.001	7.815	0.881	0.636

## Discussion

The main function of the mesenteric vein is to transport nutrients via small intestine absorption, belonging to the mixed blood. Once there is a mesenteric vein thrombosis, not only is the transportation function damaged, but the patient will also have different degrees of intestinal ischaemia. However, due to the superior mesenteric artery and branch blood vessels that supply the higher intestine [7], patients with intestinal ischaemia have a longer compensatory time. Therefore, the onset of this disease in patients occurs insidiously, and because this disease has the same initial symptoms as other causes of acute abdomen, this condition is often ignored by the first doctor that the patient sees or is even even misdiagnosed, resulting in treatment and diagnostic delay and even death.

At present, overall mesenteric vein thrombosis disease is rare [8] because less than 10% of cases are caused by mesenteric vein thrombosis. The occurrence of intestinal ischaemia in patients with acute mesenteric venous thrombosis requiring emergency surgery mainly lies in whether patients experience peritoneal irritation [9, 10], circulating shock, and bloody ascites [11]. These three aspects, to a certain extent, determine whether patients need surgical treatment after admission. However, no specific biomarkers [12] have been widely used in clinical practice to reflect patients’ systemic inflammation, liver function, kidney function and other biomarkers. This study included 8 years of well-documented clinical data from grade III, GRADE A hospitals and concluded that white blood cell count was an independent risk factor for emergency enterectomy in patients with acute mesenteric venous thrombosis. This feature may have some practical value in future emergency surgical interventions. Thirty-seven of the 42 patients who underwent bowel resection had white blood cell counts above the optimal threshold.

An elevated white blood cell count does not, however, determine the severity of acute abdomen [13]. However, it has been reported that white blood cell count can be an important predictor of intestinal necrosis caused by intestinal ischaemia [14, 15]. In this study, increased white blood cell count was an independent factor associated with intestinal resection in patients with acute mesenteric thrombosis after admission. In the intestinal resection group, 54.76% (23/42) of the patients had an initial WBC count greater than 12.87 × 10^9/L on admission. Although the accumulation of inflammatory factors in the early stage of thrombosis would lead to a slight increase in WBC count, the further increase in WBC count might be caused by further intestinal necrosis and bacterial displacement. An increased WBC count often indicates the further development of the disease, in which the patient would then require emergency exploratory laparotomy or even enterectomy. According to Kim et al. [16], leukocyte levels in MVT patients were associated with intestinal infarction, while Nuzzo et al. [17] also reported that in acute abdomen caused by abdominal pain, an increased leukocyte count should draw attention to intestinal ischaemia and necrosis. In this study, inflammation, blood clotting function, and laboratory indices of liver and kidney function of patients with acute mesenteric venous thrombosis after admission showed no correlation with acute intestinal resection, especially in patients with diabetes who should undergo emergency intestinal resection [18], but these findings have not been confirmed in this study. This may be associated with the research sample size and incorporated into the standard. Further studies are needed to confirm these results.

The arteriovenous reflection principle [19] is confirmed in patients with acute mesenteric venous thrombosis. Venous thrombosis not only leads to bowel ischaemia, which is followed by arterial spasm, but there is a further reduction of the patient’s intestinal blood supply. However, although spasm can prevent the accumulation of interstitial fluid in patients with bowel necrosis, there is an increased amount of insufflate consumption and degradation insufflate blood supply. This principle has been demonstrated in rats [20].

In conclusion, our study found that in patients with acute mesenteric venous thrombosis, the first white blood cell count on admission is closely related to the possibility of intestinal resection surgery. In such patients, bowel and venous ischaemia artery spasm due to irreversible necrosis, as well as bacterial translocation, seems to play a corresponding role in the process, and there should be timely diagnosis to prevent further intestinal necrosis and death.

## Conclusions

This study concluded that white blood cell count was an independent risk factor for enterectomy in patients after admission. Early diagnosis, timely detection of intestinal necrosis and timely surgical resection of necrotic bowel are the priorities in the treatment of this disease, which has important positive significance for improvement of life quality of patients.

## Data Availability

No datasets were generated or analysed during the current study.
